# Phenomena of Muscular Contraction

**Published:** 1846-05

**Authors:** 


*Phenomena of Muscular Contraction.*—MM. Edouard and Ernest-Hemei Weber of Leipsic, have made experiments on this subject, of which the following is an abstract:—

"There was one difficulty against which all observers of the contractility of muscle hitherto had to contend—namely, the want of a stimulus, by the aid of which they could render the contraction of sufficiently long duration under the microscope; the spasms produced by forming and breaking the galvanic current were almost instantaneous, so that the proper phenomena of the contracted fibre could not well be distinguished. By the aid of a rotatory galvano-magnetic apparatus, M. Ed. Weber was enabled to effect a permanent contraction for an entire day in the muscles of a frog, and was, in consequence, able to observe the phenomena of contractility in all their details. Muscular fibres of a frog exposed and placed on glass in a soft and curved condition, became straight at the moment of contraction; if they were straight at first they remained so on contraction and shortened; when the contraction ceased, the fibres formed the angles described by MM. Prevost and Dumas, in a very regular manner. This phenomenon is produced by the elasticity of muscular fibres, which, when contraction has ceased, endeavour to regain their former length; but the friction against the glass on which they are placed are obstacles to their free motion. The ends of the muscular fibre then cannot separate from each other, or even remain where they first were, and the fibre is bended. MM. Prevost and Dumas have, therefore, confounded the phenomena of the relaxation of a contracted fibre with that of the contraction itself. Many authors believe that the muscular fibres became *harder* during their contraction. This opinion is erroneous; the experiments of the two Webers upon the tension of relaxed and contracted muscular fibres have proved that they become more soft and extensible during their contraction. The hardness that physiologists thought they perceived in contracted muscular fibre depends rather upon their tension, like that of the tendons with which they are connected. It is very remarkable that the degree of elasticity of muscular fibres diminishes during their contraction; their substance must undergo some great change during their contraction: from M. Weber's observations it appears that the transverse muscular striæ, as well as the interstices, become broader in extension and narrower in the contracted state of the muscle; but it is incorrect to suppose that the muscular fibres are composed of discs, which are the instruments of contraction; we must not seek the cause of contraction in the action and reaction of these pretended discs, but in that of the invisible elementary molecules of which their structure is composed."—*Dublin Medical Press.*

